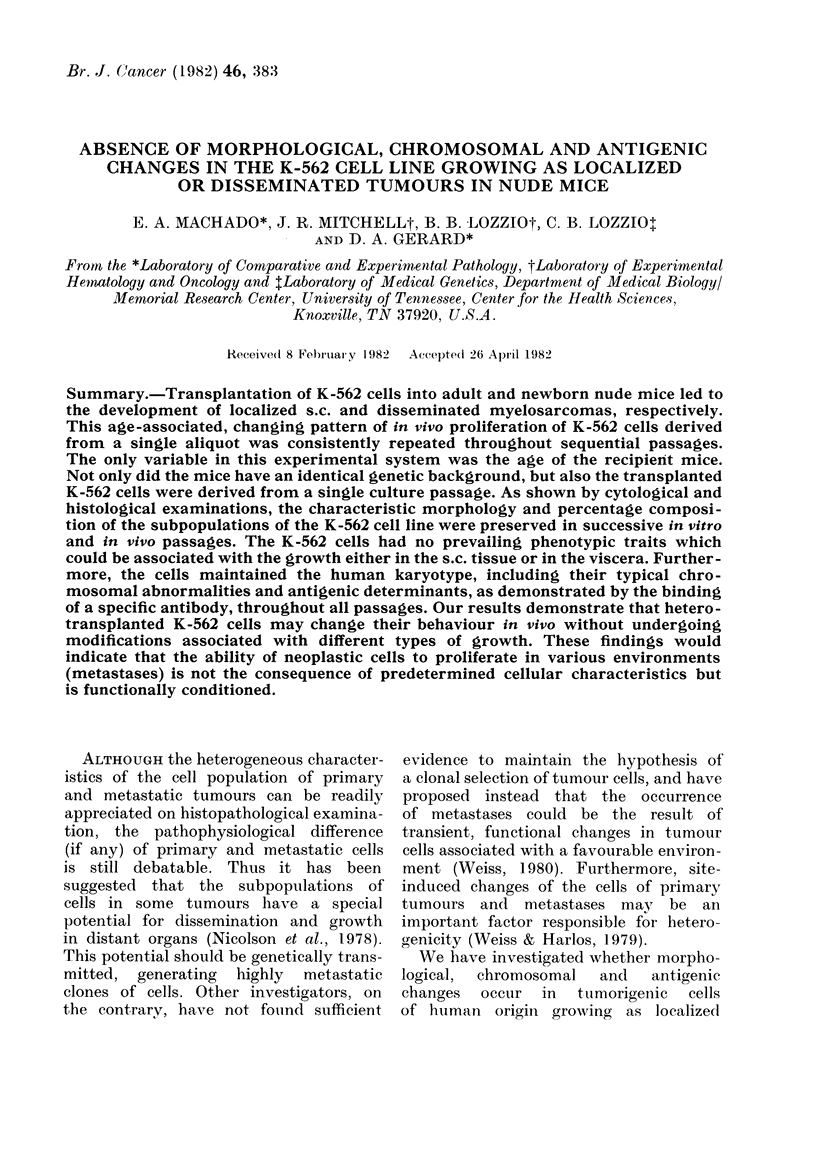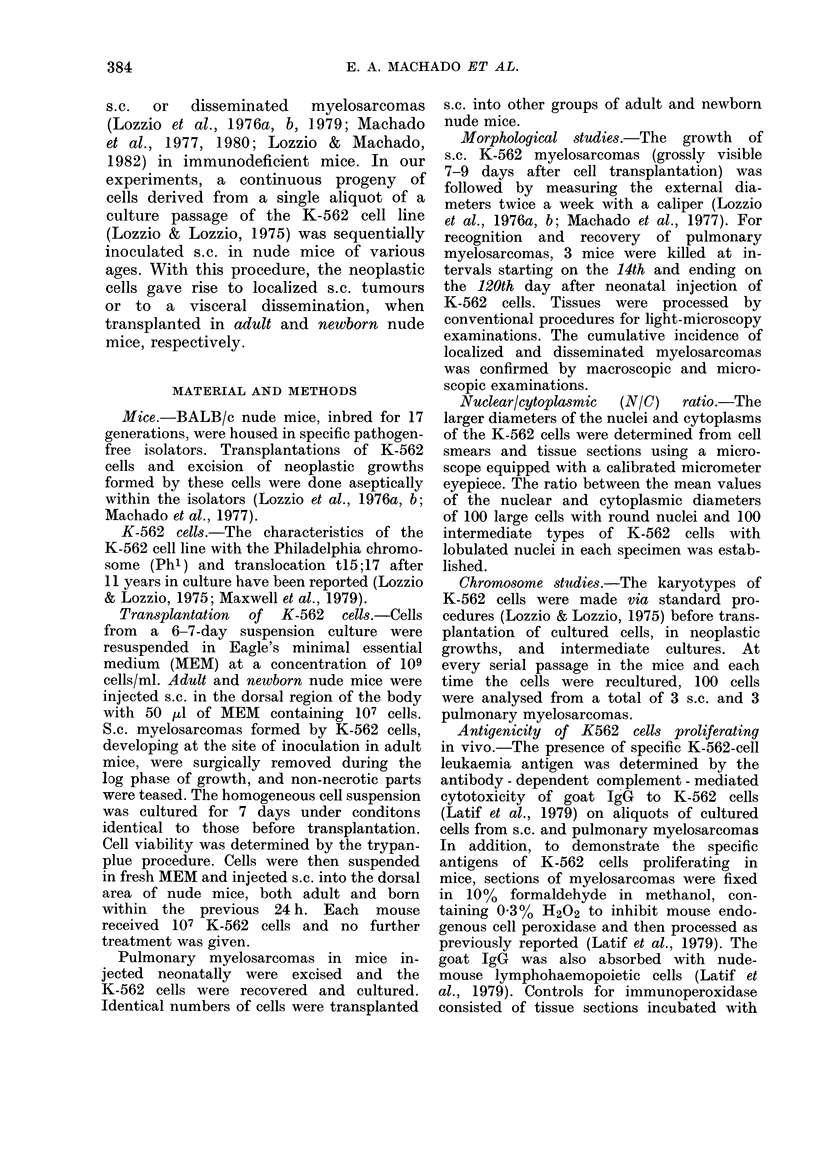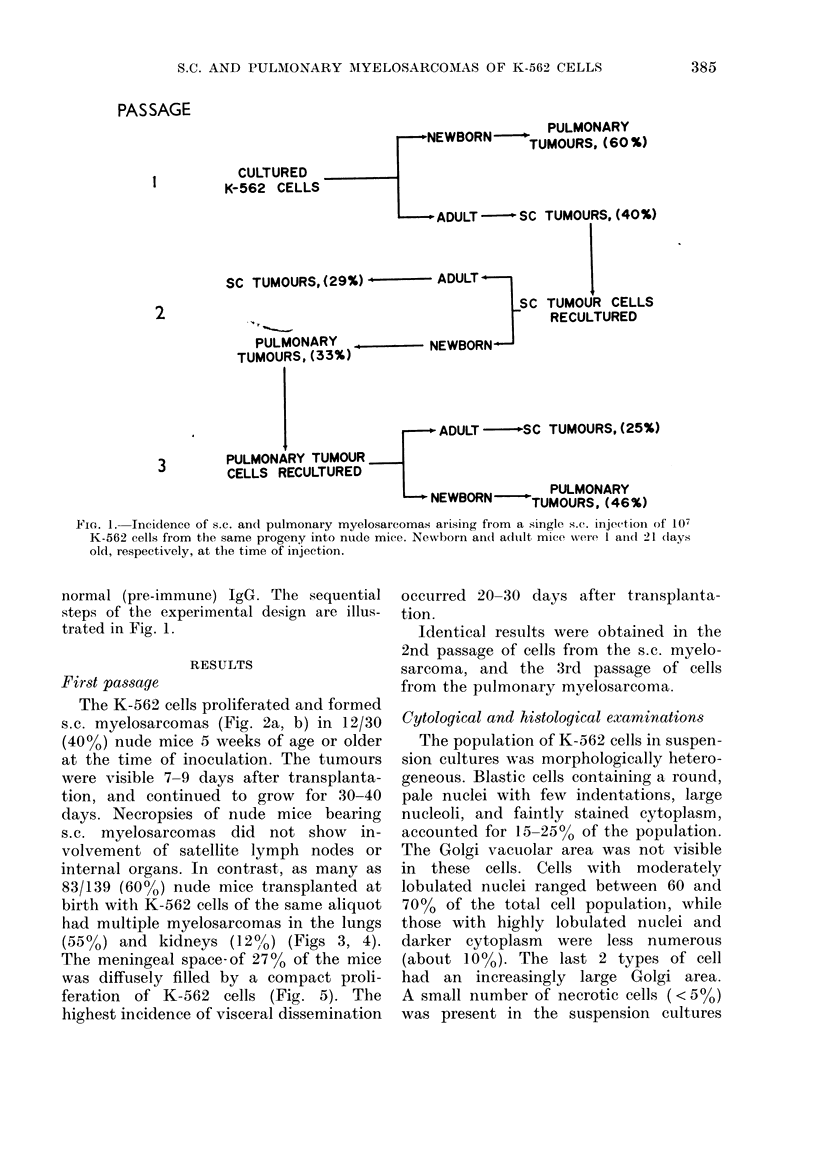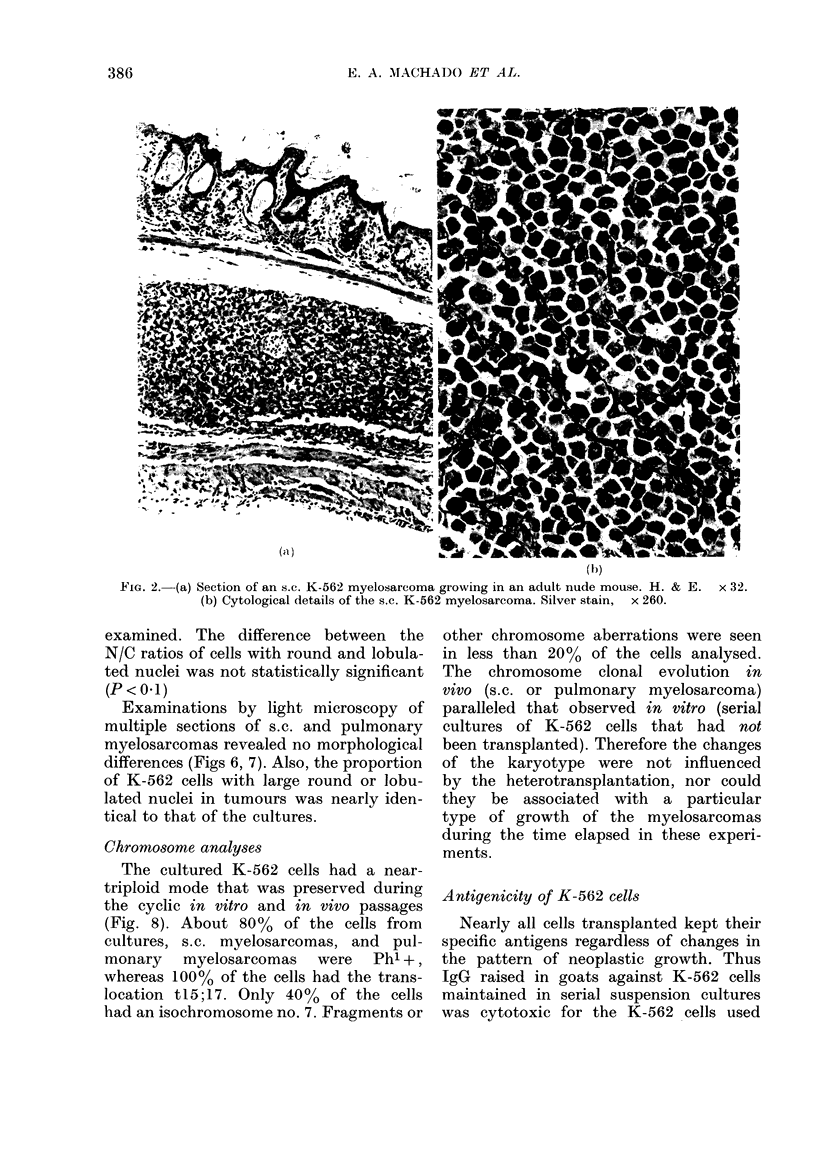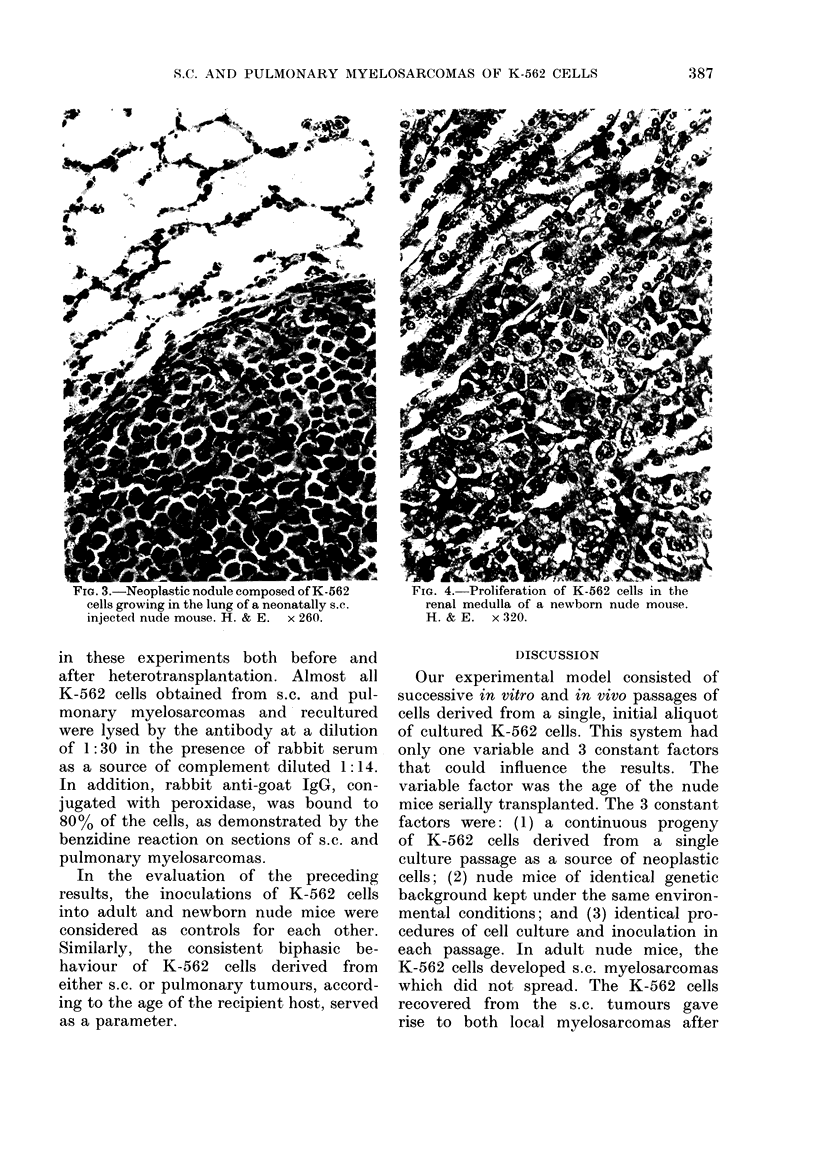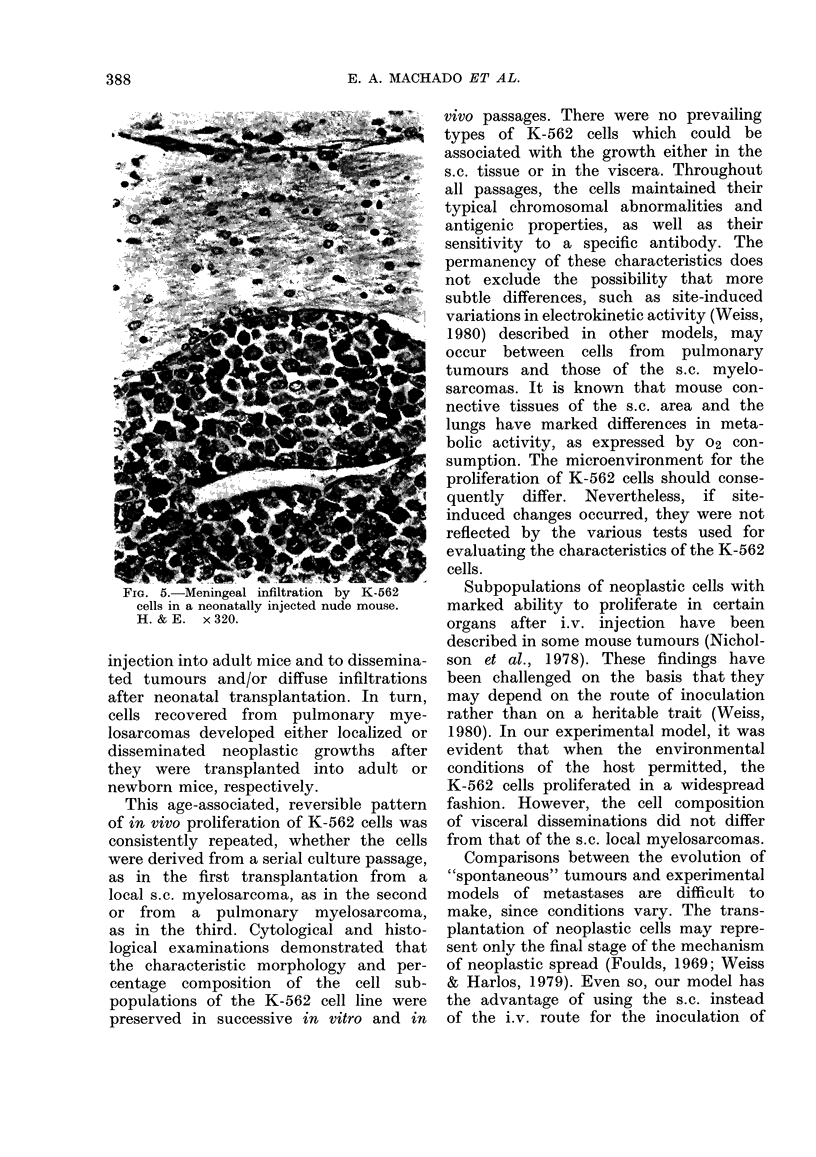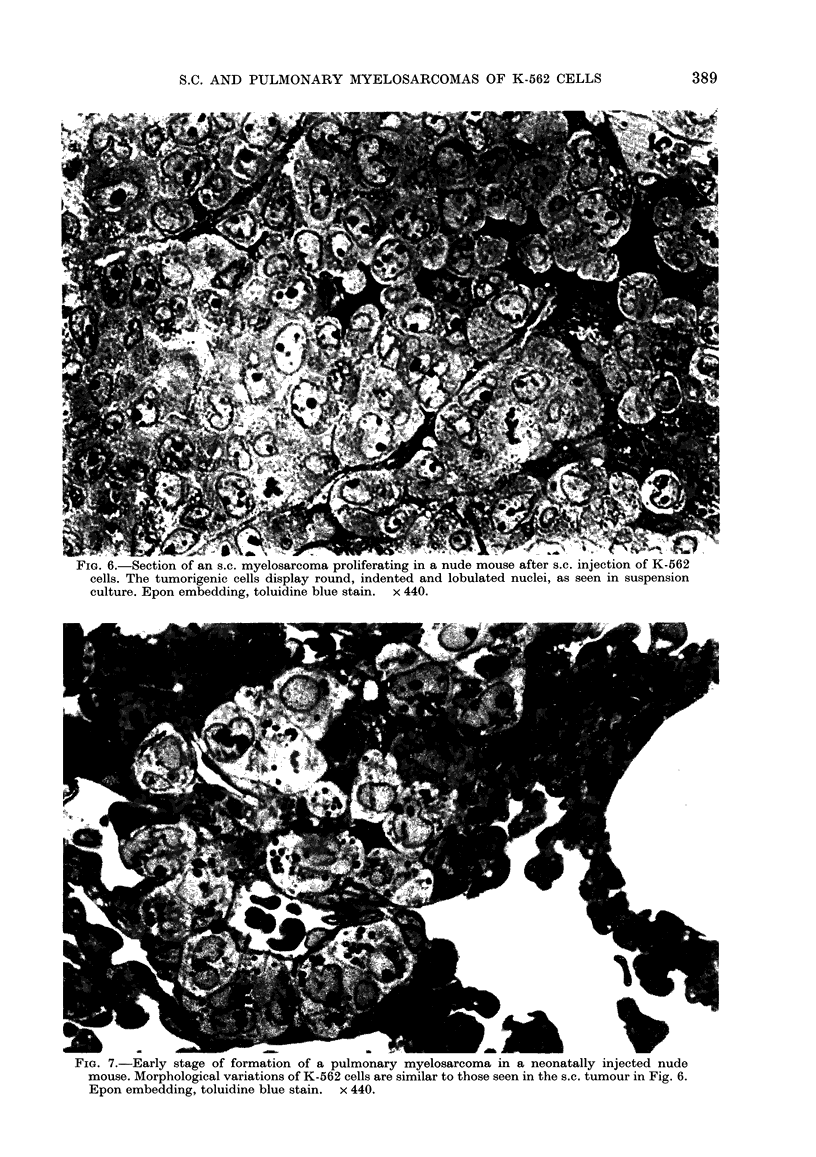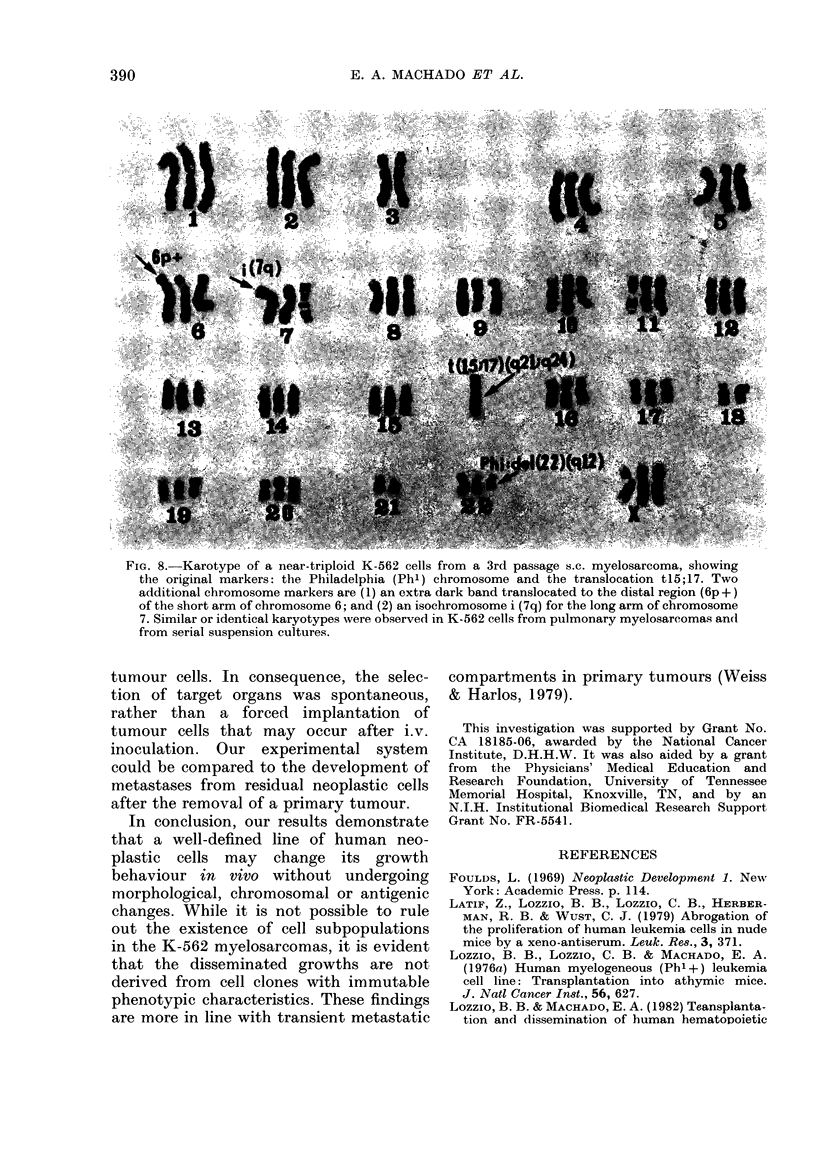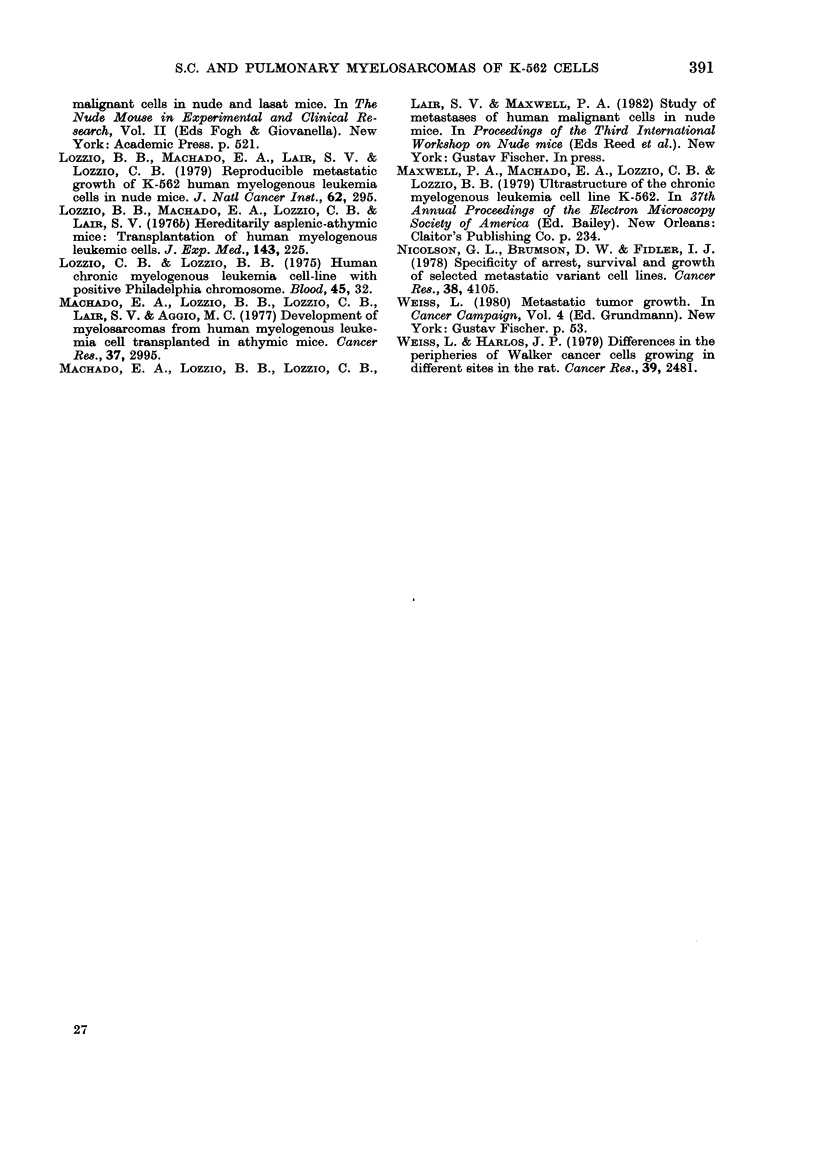# Absence of morphological, chromosomal and antigenic changes in the K-562 cell line growing as localized or disseminated tumours in nude mice.

**DOI:** 10.1038/bjc.1982.214

**Published:** 1982-09

**Authors:** E. A. Machado, J. R. Mitchell, B. B. Lozzio, C. B. Lozzio, D. A. Gerard

## Abstract

**Images:**


Br. J. Cancer (1982) 46, 383

### ABSENCE OF MORPHOLOGICAL, CHROMOSOMAL AND ANTIGENIC CHANGES IN THE K-562 CELL LINE GROWING AS LOCALIZED OR DISSEMINATED TUMOURS IN NUDE MICE

E. A. MACHADO*, J. R. MITCHELL†, B. B. LOZZIO†, C. B. LOZZIO‡ AND D. A. GERARD*

*From the *Laboratory of Comparative and Experimental Pathology, †Laboratory of Experimental Hematology and Oncology and ‡Laboratory of Medical Genetics, Department of Medical Biology/Memorial Research Center, University of Tennessee, Center for the Health Sciences, Knoxville, TN 37920, U.S.A.*

Received 8 February 1982 Accepted 26 April 1982

**Summary.—Transplantation of K-562 cells into adult and newborn nude mice led to the development of localized s.c. and disseminated myelosarcomas, respectively. This age-associated, changing pattern of *in vivo* proliferation of K-562 cells derived from a single aliquot was consistently repeated throughout sequential passages. The only variable in this experimental system was the age of the recipient mice. Not only did the mice have an identical genetic background, but also the transplanted K-562 cells were derived from a single culture passage. As shown by cytological and histological examinations, the characteristic morphology and percentage composition of the subpopulations of the K-562 cell line were preserved in successive *in vitro* and *in vivo* passages. The K-562 cells had no prevailing phenotypic traits which could be associated with the growth either in the s.c. tissue or in the viscera. Furthermore, the cells maintained the human karyotype, including their typical chromosomal abnormalities and antigenic determinants, as demonstrated by the binding of a specific antibody, throughout all passages. Our results demonstrate that heterotransplanted K-562 cells may change their behaviour *in vivo* without undergoing modifications associated with different types of growth. These findings would indicate that the ability of neoplastic cells to proliferate in various environments (metastases) is not the consequence of predetermined cellular characteristics but is functionally conditioned.**

ALTHOUGH the heterogeneous characteristics of the cell population of primary and metastatic tumours can be readily appreciated on histopathological examination, the pathophysiological difference (if any) of primary and metastatic cells is still debatable. Thus it has been suggested that the subpopulations of cells in some tumours have a special potential for dissemination and growth in distant organs (Nicolson *et al.*, 1978). This potential should be genetically transmitted, generating highly metastatic clones of cells. Other investigators, on the contrary, have not found sufficient evidence to maintain the hypothesis of a clonal selection of tumour cells, and have proposed instead that the occurrence of metastases could be the result of transient, functional changes in tumour cells associated with a favourable environment (Weiss, 1980). Furthermore, site-induced changes of the cells of primary tumours and metastases may be an important factor responsible for heterogenicity (Weiss & Harlos, 1979).

We have investigated whether morphological, chromosomal and antigenic changes occur in tumorigenic cells of human origin growing as localized s.c. or disseminated myelosarcomas (Lozzio *et al.*, 1976*a*, *b*, 1979; Machado *et al.*, 1977, 1980; Lozzio & Machado, 1982) in immunodeficient mice. In our experiments, a continuous progeny of cells derived from a single aliquot of a culture passage of the K-562 cell line (Lozzio & Lozzio, 1975) was sequentially inoculated s.c. in nude mice of various ages. With this procedure, the neoplastic cells gave rise to localized s.c. tumours or to a visceral dissemination, when transplanted in *adult* and *newborn* nude mice, respectively.

### MATERIAL AND METHODS

*Mice.*—BALB/c nude mice, inbred for 17 generations, were housed in specific pathogen-free isolators. Transplantations of K-562 cells and excision of neoplastic growths formed by these cells were done aseptically within the isolators (Lozzio *et al.*, 1976*a*, *b*; Machado *et al.*, 1977).

*K-562 cells.*—The characteristics of the K-562 cell line with the Philadelphia chromosome ($Ph^1$) and translocation t15;17 after 11 years in culture have been reported (Lozzio & Lozzio, 1975; Maxwell *et al.*, 1979).

*Transplantation of K-562 cells.*—Cells from a 6–7-day suspension culture were resuspended in Eagle's minimal essential medium (MEM) at a concentration of $10^9$ cells/ml. *Adult* and *newborn* nude mice were injected s.c. in the dorsal region of the body with 50 $\mu$l of MEM containing $10^7$ cells. S.c. myelosarcomas formed by K-562 cells, developing at the site of inoculation in adult mice, were surgically removed during the log phase of growth, and non-necrotic parts were teased. The homogeneous cell suspension was cultured for 7 days under conditons identical to those before transplantation. Cell viability was determined by the trypan-plue procedure. Cells were then suspended in fresh MEM and injected s.c. into the dorsal area of nude mice, both adult and born within the previous 24 h. Each mouse received $10^7$ K-562 cells and no further treatment was given.

Pulmonary myelosarcomas in mice injected neonatally were excised and the K-562 cells were recovered and cultured. Identical numbers of cells were transplanted s.c. into other groups of adult and newborn nude mice.

*Morphological studies.*—The growth of s.c. K-562 myelosarcomas (grossly visible 7–9 days after cell transplantation) was followed by measuring the external diameters twice a week with a caliper (Lozzio *et al.*, 1976*a*, *b*; Machado *et al.*, 1977). For recognition and recovery of pulmonary myelosarcomas, 3 mice were killed at intervals starting on the *14th* and ending on the *120th* day after neonatal injection of K-562 cells. Tissues were processed by conventional procedures for light-microscopy examinations. The cumulative incidence of localized and disseminated myelosarcomas was confirmed by macroscopic and microscopic examinations.

*Nuclear/cytoplasmic (N/C) ratio.*—The larger diameters of the nuclei and cytoplasms of the K-562 cells were determined from cell smears and tissue sections using a microscope equipped with a calibrated micrometer eyepiece. The ratio between the mean values of the nuclear and cytoplasmic diameters of 100 large cells with round nuclei and 100 intermediate types of K-562 cells with lobulated nuclei in each specimen was established.

*Chromosome studies.*—The karyotypes of K-562 cells were made *via* standard procedures (Lozzio & Lozzio, 1975) before transplantation of cultured cells, in neoplastic growths, and intermediate cultures. At every serial passage in the mice and each time the cells were recultured, 100 cells were analysed from a total of 3 s.c. and 3 pulmonary myelosarcomas.

*Antigenicity of K562 cells proliferating* in vivo.—The presence of specific K-562-cell leukaemia antigen was determined by the antibody - dependent complement - mediated cytotoxicity of goat IgG to K-562 cells (Latif *et al.*, 1979) on aliquots of cultured cells from s.c. and pulmonary myelosarcomas In addition, to demonstrate the specific antigens of K-562 cells proliferating in mice, sections of myelosarcomas were fixed in 10% formaldehyde in methanol, containing 0·3% $H_2O_2$ to inhibit mouse endogenous cell peroxidase and then processed as previously reported (Latif *et al.*, 1979). The goat IgG was also absorbed with nude-mouse lymphohaemopoietic cells (Latif *et al.*, 1979). Controls for immunoperoxidase consisted of tissue sections incubated with

FIG. 1.—Incidence of s.c. and pulmonary myelosarcomas arising from a single s.c. injection of $10^7$ K-562 cells from the same progeny into nude mice. Newborn and adult mice were 1 and 21 days old, respectively, at the time of injection.

normal (pre-immune) IgG. The sequential steps of the experimental design are illustrated in Fig. 1.

### RESULTS

*First passage*

The K-562 cells proliferated and formed s.c. myelosarcomas (Fig. 2a, b) in 12/30 (40%) nude mice 5 weeks of age or older at the time of inoculation. The tumours were visible 7–9 days after transplantation, and continued to grow for 30–40 days. Necropsies of nude mice bearing s.c. myelosarcomas did not show involvement of satellite lymph nodes or internal organs. In contrast, as many as 83/139 (60%) nude mice transplanted at birth with K-562 cells of the same aliquot had multiple myelosarcomas in the lungs (55%) and kidneys (12%) (Figs 3, 4). The meningeal space of 27% of the mice was diffusely filled by a compact proliferation of K-562 cells (Fig. 5). The highest incidence of visceral dissemination occurred 20–30 days after transplantation.

Identical results were obtained in the 2nd passage of cells from the s.c. myelosarcoma, and the 3rd passage of cells from the pulmonary myelosarcoma.

*Cytological and histological examinations*

The population of K-562 cells in suspension cultures was morphologically heterogeneous. Blastic cells containing a round, pale nuclei with few indentations, large nucleoli, and faintly stained cytoplasm, accounted for 15–25% of the population. The Golgi vacuolar area was not visible in these cells. Cells with moderately lobulated nuclei ranged between 60 and 70% of the total cell population, while those with highly lobulated nuclei and darker cytoplasm were less numerous (about 10%). The last 2 types of cell had an increasingly large Golgi area. A small number of necrotic cells (< 5%) was present in the suspension cultures

FIG. 2.—(a) Section of an s.c. K-562 myelosarcoma growing in an adult nude mouse. H. & E. × 32. (b) Cytological details of the s.c. K-562 myelosarcoma. Silver stain, × 260.

examined. The difference between the N/C ratios of cells with round and lobulated nuclei was not statistically significant ($P < 0{\cdot}1$)

Examinations by light microscopy of multiple sections of s.c. and pulmonary myelosarcomas revealed no morphological differences (Figs 6, 7). Also, the proportion of K-562 cells with large round or lobulated nuclei in tumours was nearly identical to that of the cultures.

*Chromosome analyses*

The cultured K-562 cells had a near-triploid mode that was preserved during the cyclic *in vitro* and *in vivo* passages (Fig. 8). About 80% of the cells from cultures, s.c. myelosarcomas, and pulmonary myelosarcomas were $Ph^1+$, whereas 100% of the cells had the translocation t15;17. Only 40% of the cells had an isochromosome no. 7. Fragments or other chromosome aberrations were seen in less than 20% of the cells analysed. The chromosome clonal evolution *in vivo* (s.c. or pulmonary myelosarcoma) paralleled that observed *in vitro* (serial cultures of K-562 cells that had *not* been transplanted). Therefore the changes of the karyotype were not influenced by the heterotransplantation, nor could they be associated with a particular type of growth of the myelosarcomas during the time elapsed in these experiments.

*Antigenicity of K-562 cells*

Nearly all cells transplanted kept their specific antigens regardless of changes in the pattern of neoplastic growth. Thus IgG raised in goats against K-562 cells maintained in serial suspension cultures was cytotoxic for the K-562 cells used

FIG. 3.—Neoplastic nodule composed of K-562 cells growing in the lung of a neonatally s.c. injected nude mouse. H. & E. ×260.

FIG. 4.—Proliferation of K-562 cells in the renal medulla of a newborn nude mouse. H. & E. ×320.

in these experiments both before and after heterotransplantation. Almost all K-562 cells obtained from s.c. and pulmonary myelosarcomas and recultured were lysed by the antibody at a dilution of 1:30 in the presence of rabbit serum as a source of complement diluted 1:14. In addition, rabbit anti-goat IgG, conjugated with peroxidase, was bound to 80% of the cells, as demonstrated by the benzidine reaction on sections of s.c. and pulmonary myelosarcomas.

In the evaluation of the preceding results, the inoculations of K-562 cells into adult and newborn nude mice were considered as controls for each other. Similarly, the consistent biphasic behaviour of K-562 cells derived from either s.c. or pulmonary tumours, according to the age of the recipient host, served as a parameter.

### DISCUSSION

Our experimental model consisted of successive *in vitro* and *in vivo* passages of cells derived from a single, initial aliquot of cultured K-562 cells. This system had only one variable and 3 constant factors that could influence the results. The variable factor was the age of the nude mice serially transplanted. The 3 constant factors were: (1) a continuous progeny of K-562 cells derived from a single culture passage as a source of neoplastic cells; (2) nude mice of identical genetic background kept under the same environmental conditions; and (3) identical procedures of cell culture and inoculation in each passage. In adult nude mice, the K-562 cells developed s.c. myelosarcomas which did not spread. The K-562 cells recovered from the s.c. tumours gave rise to both local myelosarcomas after

FIG. 5.—Meningeal infiltration by K-562 cells in a neonatally injected nude mouse. H. & E. ×320.

injection into adult mice and to disseminated tumours and/or diffuse infiltrations after neonatal transplantation. In turn, cells recovered from pulmonary myelosarcomas developed either localized or disseminated neoplastic growths after they were transplanted into adult or newborn mice, respectively.

This age-associated, reversible pattern of *in vivo* proliferation of K-562 cells was consistently repeated, whether the cells were derived from a serial culture passage, as in the first transplantation from a local s.c. myelosarcoma, as in the second or from a pulmonary myelosarcoma, as in the third. Cytological and histological examinations demonstrated that the characteristic morphology and percentage composition of the cell subpopulations of the K-562 cell line were preserved in successive *in vitro* and *in vivo* passages. There were no prevailing types of K-562 cells which could be associated with the growth either in the s.c. tissue or in the viscera. Throughout all passages, the cells maintained their typical chromosomal abnormalities and antigenic properties, as well as their sensitivity to a specific antibody. The permanency of these characteristics does not exclude the possibility that more subtle differences, such as site-induced variations in electrokinetic activity (Weiss, 1980) described in other models, may occur between cells from pulmonary tumours and those of the s.c. myelosarcomas. It is known that mouse connective tissues of the s.c. area and the lungs have marked differences in metabolic activity, as expressed by $O_2$ consumption. The microenvironment for the proliferation of K-562 cells should consequently differ. Nevertheless, if site-induced changes occurred, they were not reflected by the various tests used for evaluating the characteristics of the K-562 cells.

Subpopulations of neoplastic cells with marked ability to proliferate in certain organs after i.v. injection have been described in some mouse tumours (Nicholson *et al.*, 1978). These findings have been challenged on the basis that they may depend on the route of inoculation rather than on a heritable trait (Weiss, 1980). In our experimental model, it was evident that when the environmental conditions of the host permitted, the K-562 cells proliferated in a widespread fashion. However, the cell composition of visceral disseminations did not differ from that of the s.c. local myelosarcomas.

Comparisons between the evolution of "spontaneous" tumours and experimental models of metastases are difficult to make, since conditions vary. The transplantation of neoplastic cells may represent only the final stage of the mechanism of neoplastic spread (Foulds, 1969; Weiss & Harlos, 1979). Even so, our model has the advantage of using the s.c. instead of the i.v. route for the inoculation of

FIG. 6.—Section of an s.c. myelosarcoma proliferating in a nude mouse after s.c. injection of K-562 cells. The tumorigenic cells display round, indented and lobulated nuclei, as seen in suspension culture. Epon embedding, toluidine blue stain. ×440.

FIG. 7.—Early stage of formation of a pulmonary myelosarcoma in a neonatally injected nude mouse. Morphological variations of K-562 cells are similar to those seen in the s.c. tumour in Fig. 6. Epon embedding, toluidine blue stain. ×440.

FIG. 8.—Karotype of a near-triploid K-562 cells from a 3rd passage s.c. myelosarcoma, showing the original markers: the Philadelphia (Ph[1]) chromosome and the translocation t15;17. Two additional chromosome markers are (1) an extra dark band translocated to the distal region (6p+) of the short arm of chromosome 6; and (2) an isochromosome i (7q) for the long arm of chromosome 7. Similar or identical karyotypes were observed in K-562 cells from pulmonary myelosarcomas and from serial suspension cultures.

tumour cells. In consequence, the selection of target organs was spontaneous, rather than a forced implantation of tumour cells that may occur after i.v. inoculation. Our experimental system could be compared to the development of metastases from residual neoplastic cells after the removal of a primary tumour.

In conclusion, our results demonstrate that a well-defined line of human neoplastic cells may change its growth behaviour *in vivo* without undergoing morphological, chromosomal or antigenic changes. While it is not possible to rule out the existence of cell subpopulations in the K-562 myelosarcomas, it is evident that the disseminated growths are not derived from cell clones with immutable phenotypic characteristics. These findings are more in line with transient metastatic compartments in primary tumours (Weiss & Harlos, 1979).

This investigation was supported by Grant No. CA 18185-06, awarded by the National Cancer Institute, D.H.H.W. It was also aided by a grant from the Physicians' Medical Education and Research Foundation, University of Tennessee Memorial Hospital, Knoxville, TN, and by an N.I.H. Institutional Biomedical Research Support Grant No. FR-5541.

### REFERENCES

FOULDS, L. (1969) *Neoplastic Development 1.* New York: Academic Press. p. 114.

LATIF, Z., LOZZIO, B. B., LOZZIO, C. B., HERBERMAN, R. B. & WUST, C. J. (1979) Abrogation of the proliferation of human leukemia cells in nude mice by a xeno-antiserum. *Leuk. Res.*, **3**, 371.

LOZZIO, B. B., LOZZIO, C. B. & MACHADO, E. A. (1976*a*) Human myelogeneous (Ph[1]+) leukemia cell line: Transplantation into athymic mice. *J. Natl Cancer Inst.*, **56**, 627.

LOZZIO, B. B. & MACHADO, E. A. (1982) Teansplantation and dissemination of human hematopoietic malignant cells in nude and lasat mice. In *The Nude Mouse in Experimental and Clinical Research*, Vol. II (Eds Fogh & Giovanella). New York: Academic Press. p. 521.

LOZZIO, B. B., MACHADO, E. A., LAIR, S. V. & LOZZIO, C. B. (1979) Reproducible metastatic growth of K-562 human myelogenous leukemia cells in nude mice. *J. Natl Cancer Inst.*, **62**, 295.

LOZZIO, B. B., MACHADO, E. A., LOZZIO, C. B. & LAIR, S. V. (1976*b*) Hereditarily asplenic-athymic mice: Transplantation of human myelogenous leukemic cells. *J. Exp. Med.*, **143**, 225.

LOZZIO, C. B. & LOZZIO, B. B. (1975) Human chronic myelogenous leukemia cell-line with positive Philadelphia chromosome. *Blood*, **45**, 32.

MACHADO, E. A., LOZZIO, B. B., LOZZIO, C. B., LAIR, S. V. & AGGIO, M. C. (1977) Development of myelosarcomas from human myelogenous leukemia cell transplanted in athymic mice. *Cancer Res.*, **37**, 2995.

MACHADO, E. A., LOZZIO, B. B., LOZZIO, C. B., LAIR, S. V. & MAXWELL, P. A. (1982) Study of metastases of human malignant cells in nude mice. In *Proceedings of the Third International Workshop on Nude mice* (Eds Reed *et al.*). New York: Gustav Fischer. In press.

MAXWELL, P. A., MACHADO, E. A., LOZZIO, C. B. & LOZZIO, B. B. (1979) Ultrastructure of the chronic myelogenous leukemia cell line K-562. In *37th Annual Proceedings of the Electron Microscopy Society of America* (Ed. Bailey). New Orleans: Claitor's Publishing Co. p. 234.

NICOLSON, G. L., BRUMSON, D. W. & FIDLER, I. J. (1978) Specificity of arrest, survival and growth of selected metastatic variant cell lines. *Cancer Res.*, **38**, 4105.

WEISS, L. (1980) Metastatic tumor growth. In *Cancer Campaign*, Vol. 4 (Ed. Grundmann). New York: Gustav Fischer. p. 53.

WEISS, L. & HARLOS, J. P. (1979) Differences in the peripheries of Walker cancer cells growing in different sites in the rat. *Cancer Res.*, **39**, 2481.